# Long-term exposure to a ‘safe’ dose of bisphenol A reduced protein acetylation in adult rat testes

**DOI:** 10.1038/srep40337

**Published:** 2017-01-09

**Authors:** Zhuo Chen, Xuezhi Zuo, Dongliang He, Shibin Ding, Fangyi Xu, Huiqin Yang, Xin Jin, Ying Fan, Li Ying, Chong Tian, Chenjiang Ying

**Affiliations:** 1 Department of Nutrition and Food Hygiene, School of Public Health, Tongji Medical College, Huazhong University of Science and Technology, 13 Hangkong Road, Wuhan 430030, PR China; 2 MOE Key Lab of Environment and Health, School of Public Health, Tongji Medical College, Huazhong University of Science & Technology, 13 Hangkong Road, Wuhan, 430030, PR China; 3 Department of Clinical Nutrition, Tongji Hospital, Huazhong University of Science and Technology, Wuhan 430030, PR China; 4 School of Stomatology, Wenzhou Medical University, Wenzhou 325003, PR China; 5 School of Nursing, Tongji Medical College, Huazhong University of Science and Technology, 13 Hangkong Road, Wuhan 430030, PR China

## Abstract

Bisphenol A (BPA), a typical environmental endocrine-disrupting chemical, induces epigenetic inheritance. Whether histone acetylation plays a role in these effects of BPA is largely unknown. Here, we investigated histone acetylation in male rats after long-term exposure to a ‘safe’ dose of BPA. Twenty adult male rats received either BPA (50 μg/kg·bw/day) or a vehicle diet for 35 weeks. Decreased protein lysine-acetylation levels at approximately ~17 kDa and ~25 kDa, as well as decreased histone acetylation of H3K9, H3K27 and H4K12, were detected by Western blot analysis of testes from the treated rats compared with controls. Additionally, increased protein expression of deacetylase Sirt1 and reduced binding of Sirt1, together with increased binding of estrogen receptor β (ERβ) to caveolin-1 (Cav-1), a structural protein component of caveolar membranes, were detected in treated rats compared with controls. Moreover, decreased acetylation of Cav-1 was observed in the treated rats for the first time. Our study showed that long-term exposure to a ‘safe’ dose of BPA reduces histone acetylation in the male reproductive system, which may be related to the phenotypic paternal-to-offspring transmission observed in our previous study. The evidence also suggested that these epigenetic effects may be meditated by Sirt1 via competition with ERβ for binding to Cav-1.

BPA (Bisphenol A), a high-production volume chemical common in food containers and packages, is a typical EDC (environmental endocrine-disrupting chemical). Although endocrine disrupting chemicals exert effects at low doses^1^, disparities remain in the estimated TDI (tolerable daily intake) of BPA. Initially, the U.S. EPA (Environmental Protection Agency) and the EFSA (European Food Safety Agency) established the TDI of BPA at <50 μg/kg·bw/day^2^. More recent studies have shown that BPA can exert significant effects at this ‘safe’ dose^3^,^4^. After careful evaluation, the ESFA down-regulated the TDI of BPA to 4 μg/kg·bw/day in 2015, yet the U.S. EPA retained the old standard. However, the ‘safe’ dose of BPA remains controversial. As most humans are exposed to low-dose BPA on a daily basis^5^, it is important to determine the health effects of long-term exposure to a ‘safe’ dose of BPA.

Paternal BPA exposure disrupts male rat fertility and alters functions in the F1 generation^6^,^7^,^8^,^9^. In contrast to maternal exposure, paternal exposure does not involve any interaction with the litter (infant–maternal interactions). Therefore, any transmission of traits to the offspring must have been programmed into their germ cells^10^. Epigenetic inheritance is involved in the transgenerational effects of bisphenol A^11^. For example, Mao *et al*. found that paternal low-dose BPA exposure can induce pancreatic impairment in offspring via DNA methylation from the paternal germ cell^12^. Alterations in histone acetylation, an epigenetic change that is transmitted to subsequent generations, were also reported to be induced by BPA in both *in vivo* and *in vitro* studies^13^,^14^,^15^. Moreover, histone acetylation is vital for the replacement of histones by protamines and plays a critical role in protecting sperm DNA^16^. In addition, reduced histone acetylation contributes to chromatin condensing, abnormal protamine replacement and gene silencing^17^. These data suggest that histone acetylation may participate in the disruption of male rat fertility and epigenetic inheritance following exposure to BPA. However, few studies have explored histone acetylation alteration by BPA in the male reproductive system.

Sirt1, one of the best known class III histone deacetylases (HDACs), is essential for germ cell differentiation and fecundity in mice^18^. A recent study found that Sirt1 activity was inhibited by caveolin-1 (Cav-1), which is recruited it to its scaffolding domain in caveolae^19^, a membrane structure where receptors and kinases related to signal transduction are highly enriched^20^,^21^. In addition, Cav-1 also binds with estrogen receptors (ER), including ERα, ERβ, and the G protein-coupled estrogen receptor (GPER)^22^,^23^, and these receptors all bind to BPA with different affinities^24^,^25^. As both Sirt1 and ERs bind to Cav-1, an interplay between estrogen signaling and Sirt1 within the caveolae may exist. This could be the mechanism by which BPA generates its effects.

Therefore, the aim of this study was to determine whether long-term exposure to a ‘safe’ dose of BPA induces alteration of protein acetylation in the paternal reproductive system and to explore the possible mechanisms. To our knowledge, this is the first report demonstrating that essential molecules, including estrogen receptors, Cav-1, and Sirt1, are involved in this epigenetic modification.

## Results

### No adverse effect was detected in traditional toxicology studies in rat testes after exposure to BPA at 50 μg/kg·bw/day

**Figure 1:**
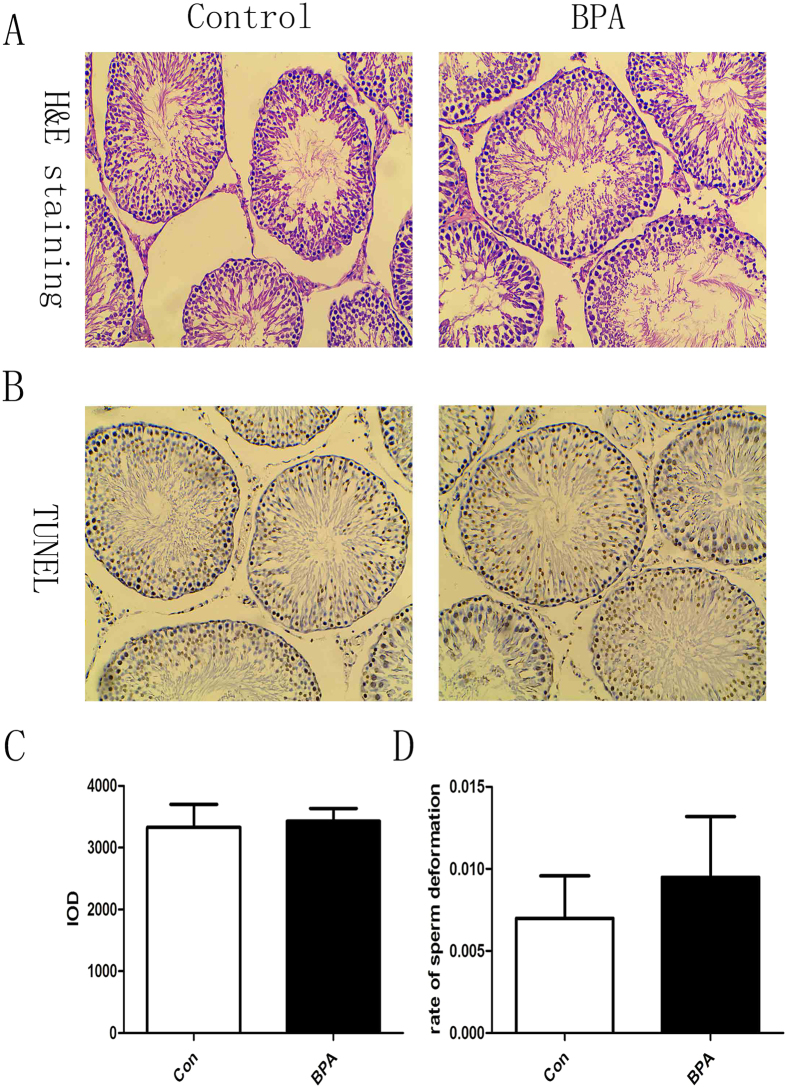
HE staining and TUNEL assay in testes and the rate of sperm deformation. HE staining and TUNEL assay were performed to assess the rates of sperm deformation (original magnification 200×). (**A**) HE staining of the testes. The majority of cells are granule cells with a dark stained nucleus. No obvious difference between the two groups was observed. (**B**) TUNEL of the testes. The brown cells are apoptotic cells. (**C**) The IOD number of TUNEL-positive cells was calculated by IPP (Image-Pro Plus 6.0). The results revealed no obvious difference between the two groups. (**D**) Rate of sperm deformation (1000 sperm cells were counted for each rat, n = 10). No difference was observed between the BPA-treated and control groups. Con: Control group, BPA: BPA treatment group.

BPA exposure did not affect body weight gain, food consumption and organ coefficiency (data not shown). The rat testes were harvested after 35 weeks of BPA exposure at 50 μg/kg·bw/day. HE staining was performed for histology, and TUNEL assay was performed to detect apoptosis in the testes. No obvious difference in general histological features was observed between the two groups (Fig. 1A,B). The IOD (Integral optical density) of TUNEL-positive cells also exhibited no significant difference (Fig. 1C) between the two groups. In addition, the sperm deformation rate was calculated, and no significant difference was documented (Fig. 1D).

### Exposure of 50 μg/kg·bw/day BPA decreased protein lysine acetylation levels

**Figure 2:**
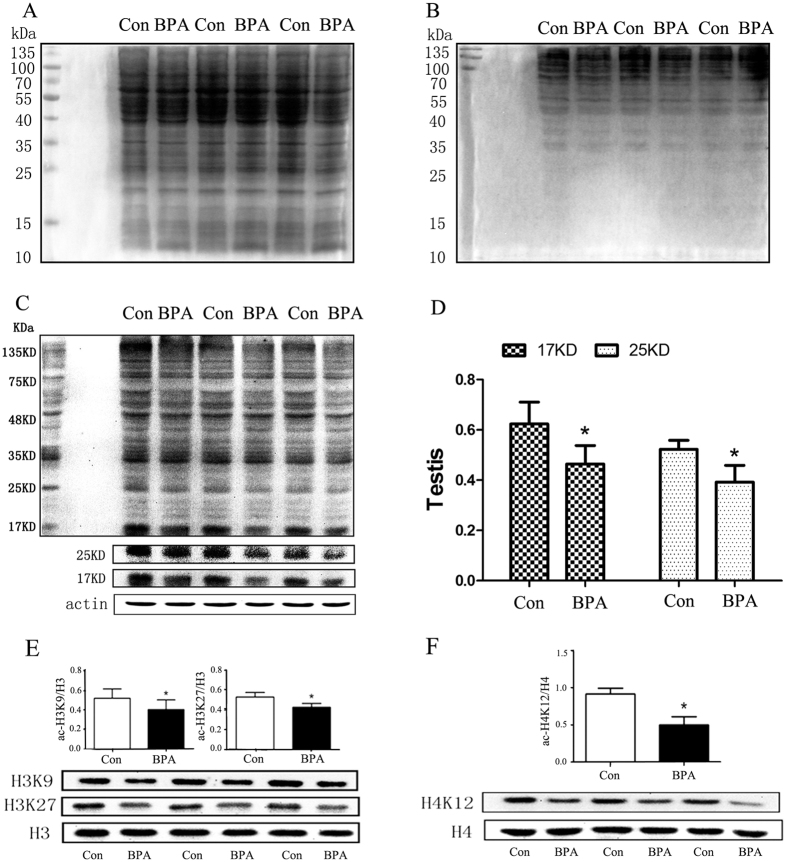
BPA exposure at 50 μg/kg · bw/day decreased protein lysine acetylation levels (n = 3). Strict quality controls were performed with Coomassie Blue staining of SDS-PAGE before (**A**) and after transfer (**B**). Lysine-acetylated proteins in the testes (**C**) were examined by Western blot analysis. Protein acetylation was decreased in the BPA-treated group compared with the control group, especially at approximately 17 KD and 25 KD (**D**). Histone acetylation levels were presented in arbitrary units using total protein of H3 and H4 as references. (**E**) H3K9 and H3K27 acetylation decreased significantly in the BPA group compared with the control group. (**F**) H4K12 acetylation decreased significantly in the BPA group compared with the control group. Cropped blots are displayed, and the contrast ratio of the 25 KD band was adjusted in (**C**) for clarity. Con: Control group, BPA: BPA treatment group.

Lysine-acetylated proteins in the testes were detected. Quality control for the experiment performance by Coomassie Blue staining is presented in Fig. 2A,B. Significant decreases in protein lysine acetylation levels (Fig. 2C) were detected in the treated rats compared with controls, typically at two areas near 17 kDa and 25 kDa (Fig. 2D). Acetylation of histones in rat testes were assayed using respective antibodies. In accordance with the obvious decreased acetylation at 17 kDa and 25 kDa, decreased levels of ac-H3K9, ac-H3K27 (*P* < 0.05; Fig. 2E), and ac-H4K12 (*P* < 0.01; Fig. 2F) were detected in the treated rats compared with controls.

### BPA treatment increased the expression of histone deacetylase Sirt1

**Figure 3:**
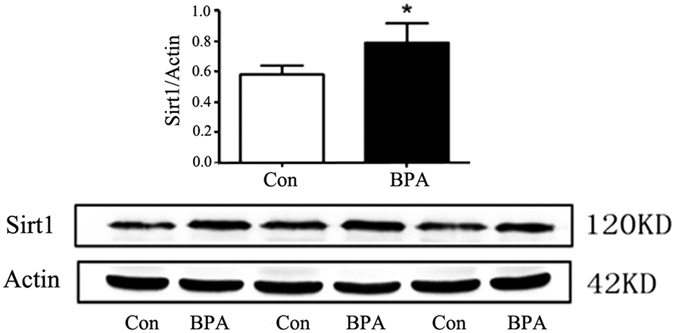
BPA treatment increased the expression of histone deacetylase Sirt1 (n = 3). Expression of the histone deacetylase Sirt1 increased significantly in the BPA group compared with the control group. Cropped blots are displayed. Data are expressed as the mean ± SEM, *P < 0.05 compared with the control group. Con: Control group, BPA: BPA treatment group.

In addition to decreased histone acetylation, we further detected a significant increase in the expression of histone deacetylase Sirt1 in the testes of BPA-treated rats compared with controls (*P* < 0.05; Fig. 3).

### BPA treatment decreased Cav-1 acetylation without altering its expression

**Figure 4:**
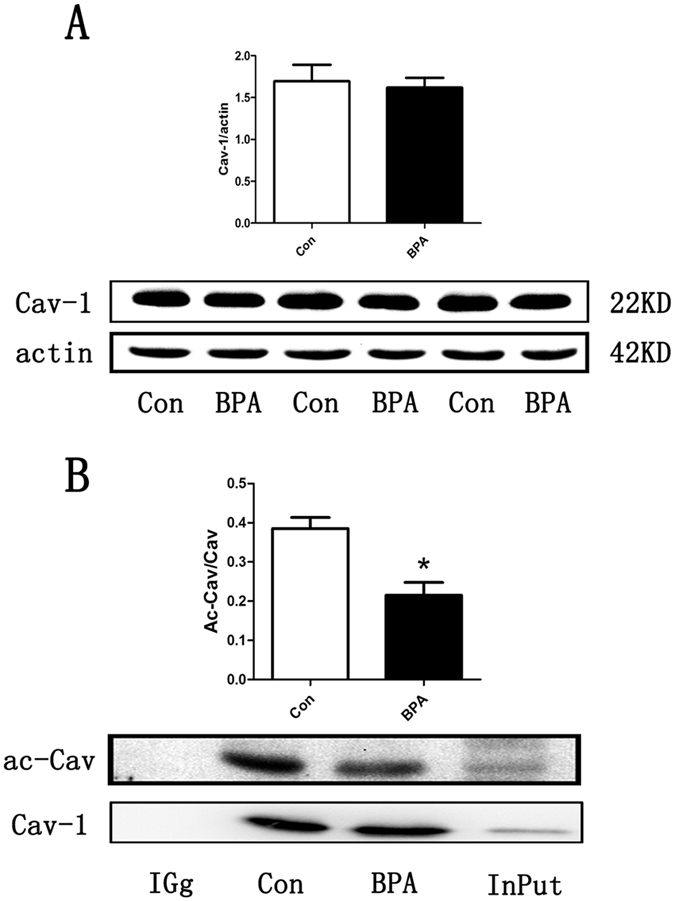
BPA treatment decreased Cav-1 acetylation without altering its expression (n = 3). (**A**) Protein expression of Cav-1. (**B**) IP results using normal IgG as the negative control and protein lysate as the positive control. Protein was precipitated with Cav-1 antibody and then immunoblotted with acetylated-lysine antibody. The results were presented in arbitrary units using beta-actin or purified Cav-1 as a reference. Data are expressed as the mean ± SEM, *P < 0.05 compared with the control group. Cropped blots are displayed, and the contrast ratio of the Ac-Cav band was adjusted in (**B**) for clarity. Con: Control group, BPA: BPA treatment group.

As shown in Fig. 4, no difference in Cav-1 protein level was observed in BPA-treated rat testes compared with the control group (Fig. 4A). IP detection demonstrated a significant reduction of acetylated Cav-1 in BPA-treated rats (*P* < 0.05; Fig. 4B).

### BPA decreased Sirt1 binding and increased ERβ binding to Cav-1

**Figure 5:**
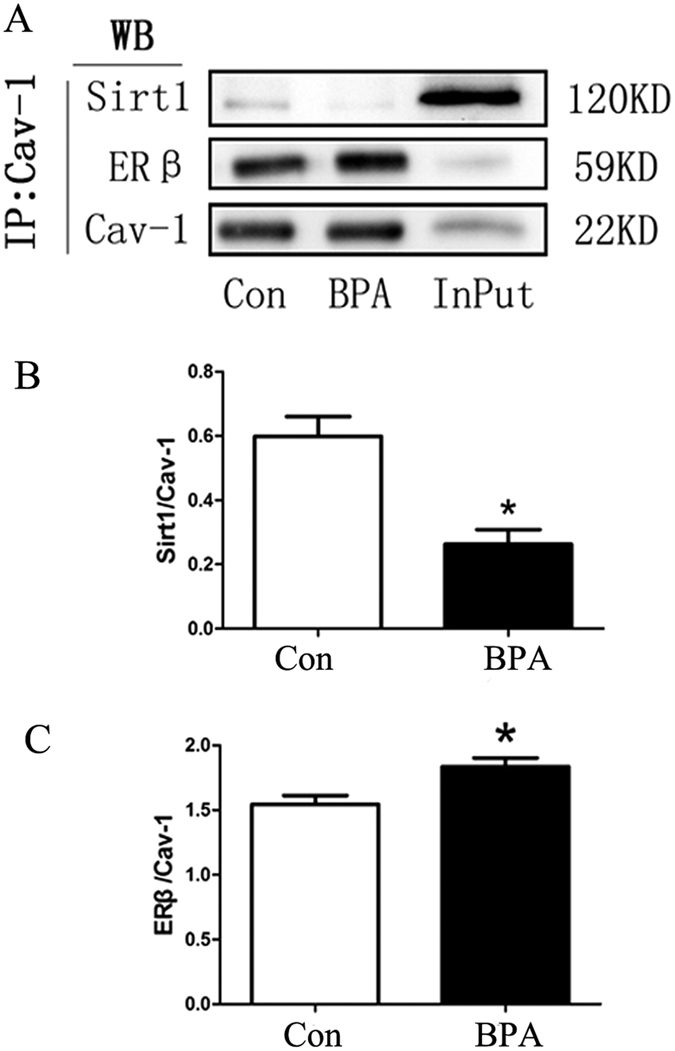
BPA decreased Sirt1 binding and increased ERβ binding to Cav-1 (n = 3). The immunoprecipitated complexes were pulled down with Cav-1 antibody and then immunoblotted with ERβ and Sirt1 antibodies. Immunoblotting results are presented in (**A**). The results were normalized according to Cav-1, and relative protein levels of Sirt1 and ERβ are presented in (**B**,**C**), respectively. Cropped blots are displayed. Data are expressed as the mean ± SEM, *P < 0.05 compared with the control group. Con: Control group, BPA: BPA treatment group.

To investigate whether BPA estrogenic effects influenced binding of Cav-1 to Sirt1 and ERs, IP detection was performed. As shown in Fig. 5, the results showed that BPA treatment decreased the binding of Sirt1 to Cav-1 (*P* < 0.05; Fig. 5B) and simultaneously increased ERβ binding to Cav-1 (*P* < 0.05; Fig. 5C) in the treatment group compared with the control group.

### Exposure of BPA at 50 μg/kg·bw/day increased ERβ expression in testes

**Figure 6:**
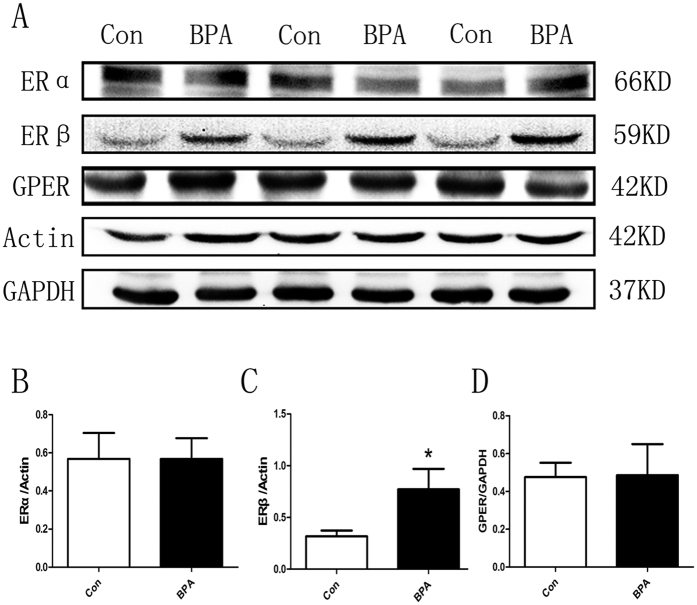
Exposure to 50 μg/kg·bw/day BPA increased ERβ expression in testes (n = 3). Western blotting bands of ERα, ERβ and GPER are presented in (**A**). The results were normalized by beta-actin or GAPDH. Relative protein levels of ERα, ERβ and GPER are presented in (**B**–**D**), respectively. Cropped blots are displayed. Data are expressed as the mean ± SEM, *P < 0.05 compared with the control group. Con: Control group, BPA: BPA treatment group.

To investigate the roles of estrogen receptors, the protein levels of estrogen receptors (ERα, ERβ and GPER) were detected by Western blot (Fig. 6). ERβ expression was remarkably elevated in the testes of BPA-treated rats compared with the control group (*P* < 0.05; Fig. 6C), whereas no significant difference in ERα or GPER expression was observed (Fig. 6B,D).

## Discussion

In the present study, decreased histone acetylation of H3K9, H3K27, and H4K12 in rat testes was observed after long-term exposure to a ‘safe’ dose of BPA compared with the control group, and the evidence suggests that this effect may be mediated by increased Sirt1 disassociation from Cav-1.

Concern is mounting regarding the environmental effects of BPA on human health, especially on reproductive toxicity. In the present study, no effect of BPA exposure on food consumption, body weight, or the relative weight of the testes or epididymis was observed (data not shown). In addition, no apparent effects were detected by HE staining, TUNEL assay or rate of sperm deformity (Fig. 1). The results were consistent with a previous study demonstrating no effects on the reproductive organs of male mice upon oral exposure of 2, 20, or 200 μg/kg BPA^26^. Our recent findings showed that low-dose BPA exposure of male rats induced spatial memory disorder and was transmitted to the offspring^9^. Here, a similar adult male rat model was used, and, compared with the control group, a remarkable reduction in acetylation of H3 (Lys9 and Lys27) and H4 at lysine 12 were detected in the testes (Fig. 2) where spermatogenesis occurs, and the majority of histones were replaced with protamines^27^. Consistent with those findings, Zhang *et al*. reported that adult male mice exposed to doses lower than the NOAEL (No Observed Adverse Effect Level) (50 mg/kg · bw/day) estimated by the US EPA affected histone H3 acetylation of the hippocampus^15^, and an *in vitro* study demonstrated that chronic exposure to 3 nM BPA during follicle culture affects histone H4K12 acetylation of germinal vesicles and metaphase II oocytes^28^. The predicted ‘safe’ dose of BPA is calculated by dividing the lowest observed adverse effect level by three safety factors (human variability, interspecies differences, and variability in toxicokinetics and toxicodynamics, ten-fold for each factor). However, treatment with BPA (10–50 μg/kg·bw/day) causes numerous effects in rodents. If the three ten-fold safety factors noted above were considered, the TDI of BPA is on the nanogram level, which can be easily achieved via daily diet exposure in humans. For example, exposure of BPA at 7.50 μg/day in adults was observed in Germany^29^ and approximately 30.0 μg/day in Taiwan^30^. In our recent report, BPA in 450 food samples representing 7 food categories (mainly raw and fresh food) collected from three geographic cities in China were detected at the nanogram level (e.g., the mean value was 7.708 ng/g fresh weight for cereals and cereal products)^31^, indicating that the daily exposure through food for adults would be at the microgram level. The present study showed that long-term exposure to a ‘safe’ dose of BPA reduced histone acetylation in the male reproductive system in rats. Although this evidence may not directly extrapolate to humans, the risk of daily exposure to BPA should be considered seriously.

Protein levels of histone deacetylase Sirt1 in rat testes were significantly increased in the BPA-treated group compared with the control group (Fig. 3) in this study. Similarly, estrogenic agents, including 17β-estradiol and the selective ligand of GPER, namely, G-1, also up-regulated Sirt1 expression^32^. It was reported that histone acetylation was altered, and the replacement of histones with protamines was disrupted in Sirt1 KO mice^18^. Thus, BPA exposure induced a reduction of protein acetylation, and histone acetylation in rat testes is regulated via increased Sirt1 expression. Cav-1 inhibits Sirt1 by binding it to the scaffolding area^19^. Results in the present study revealed no change in the expression level of Cav-1, but a remarkable reduction in Cav-1 acetylation (Fig. 4) was observed in the treated group compared with the control group. Given that acetylation of Cav-1 is rarely reported^33^, more studies are needed to determine whether the acetylation of Cav-1 is involved in the regulation of Sirt1 levels. In addition, Cav-1 also binds with estrogen receptors^22^ and increases histone acetylation^34^,^35^. Our immunoprecipitation results revealed that BPA treatment decreased the binding of Sirt1 to Cav-1 and simultaneously increased the binding of ERβ, the most abundant ER in rat testes^36^, to Cav-1 compared with the control group (Fig. 5). Consistent with previous studies^24^, ERβ was significantly up-regulated by BPA exposure (Fig. 6), but no obvious change in ERα or GPER was observed. Our previous study found that X-linked inhibitor of apoptosis protein (XIAP) competes with eNOS for Cav-1 binding^37^. We propose that competition between Sirt1 and ERβ for Cav-1 binding may also exist, and that Sirt1 dissociation from caveolae may account for the increased level of Sirt1. Given that BPA can interact with ERβ with 10-fold increased affinity compared with ERα^24^,^38^,^39^, ERβ is likely involved, at least in part, in the BPA-induced estrogenic effect in rat testes. To our knowledge, this is the first study that has investigated the relationship of the histone acetylation status in the testes and the involvement of ERs, Sirt1 and Cav-1 in a long-term BPA exposure model. However, more studies are required to verify the underlying mechanism of BPA on histone acetylation.

## Conclusion

The present study demonstrated that long-term exposure to a ‘safe’ dose of BPA induced a reduction in protein acetylation in rat testes, and that this effect may be meditated by increased Sirt1 expression. This increase in Sirt1 levels may be caused by the dissociation of Sirt1 from Cav-1 binding via competition with ERβ for Cav-1. Our findings provide some clues regarding the underlying mechanisms for epigenetic inheritance induced by long-term low-dose BPA exposure. However, further studies are needed to clarify the mechanism.

## Methods

### Animal care

Animals were maintained in accordance with the Guidelines for the Care and Use of Laboratory Animals (Institute of Laboratory Animal Resources, National Academies Press, Washington D.C., 1996), and ethical approval was granted by the Ethics Committee of Tongji Medical College (Huazhong University of Science and Technology, Wuhan, China). Twenty adult male Wistar rats (150–180 g) were supplied by Hubei Research Center of Laboratory Animal, Wuhan, China. Animals were individually housed in special polypropylene cages with ad libitum access to food and water in an environmentally controlled room (temperature, 21 ± 1 °C; relative humidity, 60 ± 10%) maintained on a 12-h light/dark cycle (lights off from 18:00 to 06:00).

### Experimental design

After one week of acclimation, paternal animals were equally and randomly divided into a BPA (50 μg/kg·bw/day) or a control diet group. BPA (purity ≥ 99%) was obtained from Sigma Chemical Company. The details of the BPA (50 μg/kg·bw/day) treatment methods have been described previously^40^. After 35 weeks of treatment, all rats were sacrificed under anesthesia. The testes and epididymis were dissected and weighed, and the values were used to calculate the testes and epididymis coefficients. Then, one testis from each animal was immediately frozen in liquid nitrogen and stored at −80 °C for future experiments. The other testis was fixed in 4% paraformaldehyde for HE staining and TUNEL experiments.

### HE staining, TUNEL and microscope

The testes were fixed in 4% paraformaldehyde, dehydrated, and embedded in paraffin. Part of the rat testes sections were stained with hematoxylin and eosin (HE) staining, and part of the sections were used to assess apoptosis via the TUNEL (TdT-mediated dUTP Nick-End Labeling) DNA strand breaks assay (Roche, No. 11684817910). Epididymis tissues were prepared to observe abnormal sperm cells under a microscope. After being separated and weighed, the caudal epididymis was dissected and punctured followed by 30-min incubation in normal saline at 37 °C. Straws were used to withdraw some fluid, which was uniformly smeared onto a slide for observation under a microscope. Then, the rate of sperm deformation was calculated.

### SDS-PAGE and Western blot analysis

The SDS-PAGE and Western blotting procedures were performed according to manufacturer’s instructions. Rat testes samples (approximately 100 mg of each) were lysed for 2 h at 4 °C in RIPA lysis buffer (Beyotime) with appropriate phenylmethanesulfonyl fluoride (PMSF). Then, equal amounts of reduced protein (50–75 μg as needed) were resolved via SDS-PAGE and transferred to polyvinylidene fluoride (PVDF) membranes (0.45 μm, Bio-Rad) according to the method described by Amersham Biosciences. All membranes were incubated in blocking buffer (5% skim milk in 0.1% Tween-20 PBS) at 4 °C for 2 h. Subsequently, membranes were incubated in blocking buffer overnight at 4 °C containing primary antibodies purchased from Cell Signaling Technology (Billerica, MA, USA) against caveolin-1 (Cav-1), H3K9, H3K27, H4K12, Sirt1, and histones H3 and H4. The membranes were then washed three times in washing buffer (Tris-buffered saline and 0.05% Tween-20) for 10 min each time and then incubated with the respective HRP-conjugated secondary antibodies (1:5000; Beyotime, Haimen, Jiangsu, China) for 1 h at room temperature. After the membranes were washed three times again, protein expression was visualized with an ECL detection system (Syngen, Cambridge, UK) and analyzed using the Chemidoc-Quantity-One software (Bio-Rad Laboratories). Acetylated-Lysine Mouse mAb (Ac-K-103) antibody was purchased from Cell Signaling Technology (Billerica, MA, USA). ERα and ERβ antibodies were obtained from Merck Millipore (Billerica, MA, USA). GPER antibody was purchased from ABCAM. All antibodies were used according to the manufacturer’s instructions. β-actin (1:8000, Sigma–Aldrich Chemical Company) or GAPDH (1:5000, Proteintech) was used as a loading control. Experiments were performed three independent times, and representative images are presented.

### Immunoprecipitation (IP)

The total protein concentration solutions were prepared for Western blot and IP (Life technology). Equal amounts of Dynabeads^®^ and Cav-1 antibody were incubated under the same conditions, and equivalent protein solutions were used to immunoprecipitate the target antigen. All procedures were performed at room temperature. Finally, Cav-1, ERβ, and Sirt1 expression were determined by Western blot. Acetylated-Cav-1 was incubated with Acetylated-Lysine Mouse mAb (Ac-K-103) antibodies.

### Statistical analysis

Data were the means of three experiments conducted with 3 animals from each group, and the results were presented as the mean ± SEM. Statistical analysis was performed using SPSS 13.0 (SPSS, Chicago, IL). Data were compared using an unpaired Student’s *t* test. All the *P*-values were two-tailed, and differences were considered significant when *P* < 0.05.

## Additional Information

**How to cite this article**: Chen, Z. *et al*. Long-term exposure to a ‘safe’ dose of bisphenol A reduced protein acetylation in adult rat testes. *Sci. Rep.* **7**, 40337; doi: 10.1038/srep40337 (2017).

**Publisher's note:** Springer Nature remains neutral with regard to jurisdictional claims in published maps and institutional affiliations.

## Supplementary Material

Supplementary Information
