# Supplementary material for: Long-term exposure to a ‘safe’ dose of bisphenol A reduced protein acetylation in adult rat testes
Source: Sci Rep. 2017 Jan 9;7:40337. doi: 10.1038/srep40337 (PMC5220302; doi:10.1038/srep40337)

Supplementary Information

Ref: SREP-16-21056D

Long-term exposure to a 'safe' dose of bisphenol A reduced protein acetylation in adult rat testes

Zhuo Chen[1,2,3], Xuezhi Zuo[1,4], Dongliang He[2], Shibin Ding[2], Fangyi Xu[2], Huiqin Yang[2], Xin Jin[2], Ying Fan[2], Li Ying[5], Chong Tian[6], *, Chenjiang Ying[2,3,*]

Supplementary Information includes:

1. Title page
2. Original images of cropped blots

Long-term exposure to a 'safe' dose of bisphenol A reduced protein acetylation in adult rat testes

Zhuo Chen[1,2,3], Xuezhi Zuo[1,4], Dongliang He[2], Shibin Ding[2], Fangyi Xu[2], Huiqin Yang[2], Xin Jin[2], Ying Fan[2], Li Ying[5], Chong Tian[6, *], Chenjiang Ying[2,3,*]

Authors' Affiliations:

[1] The authors contribute equal to the research

[2] Department of Nutrition and Food Hygiene, School of Public Health, Tongji Medical College, Huazhong University of Science and Technology, 13 Hangkong Road, Wuhan 430030, PR China

[3] MOE Key Lab of Environment and Health, School of Public Health, Tongji Medical College, Huazhong University of Science & Technology, 13 Hangkong Road, Wuhan, 430030, PR China

[4] Department of Clinical Nutrition, Tongji Hospital, Huazhong University of Science and Technology, Wuhan 430030, PR China

[5] School of Stomatology, Wenzhou Medical University, Wenzhou 325003, PR China

[6] School of Nursing, Tongji Medical College, Huazhong University of Science and Technology, 13 Hangkong Road, Wuhan 430030, PR China

*__Corresponding Author:__

Chong Tian

Address: School of Nursing, Tongji Medical College, Huazhong University of Science and Technology, 13 Hangkong Road, Wuhan, Hubei, 430030, China

Tel.: +86-27-83692635

Fax: +86-27-83692658

E-mail: tianchong0826@hust.edu.cn

Chenjiang Ying

Address: School of Public Health, Tongji Medical College, Huazhong University of Science and Technology, 13 Hangkong Road, Wuhan, Hubei, 430030, China

Tel.: +86-27-83650523

Fax: +86-27-83693673

E-mail: yingcj@hust.edu.cn

Original images of cropped blots

Fig. 2 C

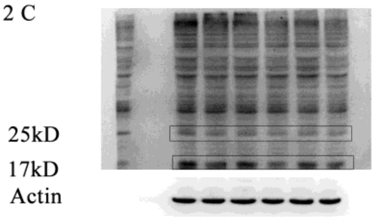

Fig. 2 E

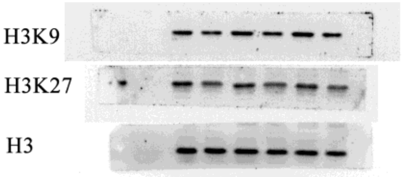

Fig. 2 F

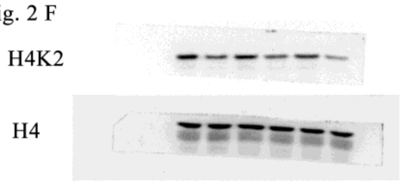

Fig. 3

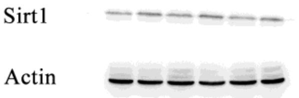

Fig. 4A

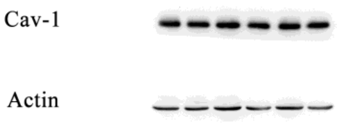

Fig. 4B

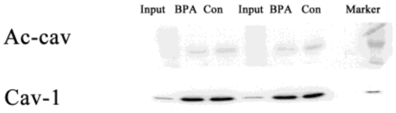

Fig. 5

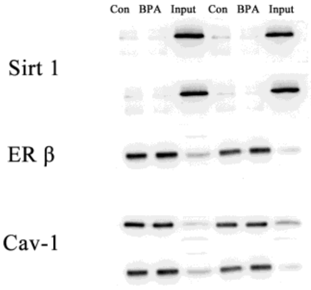

Fig. 6

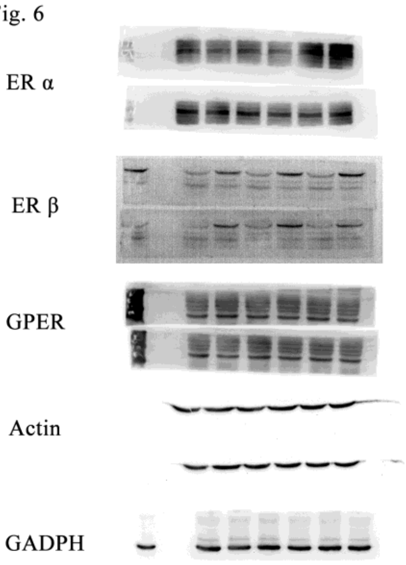

Supplement: Supplementary Information [file srep40337-s1.pdf]
